# Cyclic Hypoxia: An Update on Its Characteristics, Methods to Measure It and Biological Implications in Cancer

**DOI:** 10.3390/cancers13010023

**Published:** 2020-12-23

**Authors:** Samuel B. Bader, Mark W. Dewhirst, Ester M. Hammond

**Affiliations:** 1 Department of Oncology, The Oxford Institute for Radiation Oncology, Oxford University, Oxford OX3 7DQ, UK; samuel.bader@oncology.ox.ac.uk; 2 Radiation Oncology Department, Duke University School of Medicine, Durham, NC 27710, USA

**Keywords:** hypoxia, cyclic, intermittent, transient

## Abstract

**Simple Summary:**

The term hypoxia is used to describe biological situations where insufficient levels of oxygen exist. Hypoxia and hypoxic states can occur in a range of diseases including in cancer. Researchers have understood for numerous years that the hypoxia found in tumors leads to more aggressive and harder to treat disease and ultimately, poor patient outcome. While much research is, and has been, carried out to investigate the effects of hypoxia, this is usually done using experimental models which employ stable levels of hypoxia. However, we know that tumor hypoxia is not static but instead is rapidly changing through complex processes involving both rapid and slower fluctuations in oxygen concentrations. These dynamic changes in oxygen, known as cyclic hypoxia, are challenging to model experimentally and therefore our understanding of these processes has been limited. This review seeks to outline the known causes of cyclic hypoxia and the best ways to measure it both experimentally and clinically. How cancer cells respond to cyclic hypoxic compared to stable levels will also be discussed.

**Abstract:**

Regions of hypoxia occur in most if not all solid cancers. Although the presence of tumor hypoxia is a common occurrence, the levels of hypoxia and proportion of the tumor that are hypoxic vary significantly. Importantly, even within tumors, oxygen levels fluctuate due to changes in red blood cell flux, vascular remodeling and thermoregulation. Together, this leads to cyclic or intermittent hypoxia. Tumor hypoxia predicts for poor patient outcome, in part due to increased resistance to all standard therapies. However, it is less clear how cyclic hypoxia impacts therapy response. Here, we discuss the causes of cyclic hypoxia and, importantly, which imaging modalities are best suited to detecting cyclic vs. chronic hypoxia. In addition, we provide a comparison of the biological response to chronic and cyclic hypoxia, including how the levels of reactive oxygen species and HIF-1 are likely impacted. Together, we highlight the importance of remembering that tumor hypoxia is not a static condition and that the fluctuations in oxygen levels have significant biological consequences.

## 1. Introduction

Hypoxia is a well-established physiological feature of many solid cancers. Hypoxia occurs when tumor sub-regions have insufficient oxygen concentration to support aerobic metabolic functions. The radioresistance associated with tumour hypoxia is a result of the need for oxygen to be present during irradiation or exposure to certain cytotoxic drugs which create stable treatment-induced DNA adducts that are difficult to repair [1]. Furthermore, certain forms of DNA repair mechanisms are inhibited under hypoxic conditions [1]. Hypoxia also impedes the immune responses to tumors, via multiple mechanisms [2]. Importantly, the tumor microenvironment includes variable and fluctuating oxygen concentrations, which result in dynamic tumor oxygenation referred to as cyclic or intermittent hypoxia. Changes in signal transduction and metabolism as well as radiosensitivity occur when pO_2_ drops below 10 mmHg, so this is a convenient threshold to use to define hypoxia [3]. Hypoxia occurs in two forms: (1) when cells are experiencing hypoxia at quasi-steady state, it is referred to as “chronic”. (2) Temporal variation of pO_2_ that fluctuates above and below the 10 mmHg threshold, is known as intermittent, acute, transient, or cyclic hypoxia [4]. The objective of this review is to focus on the subject of cyclic hypoxia in human cancer, as its causes, characteristics and consequences are less defined than chronic hypoxia. The review has two parts: physiologic characterization, potential mechanisms that cause cyclic hypoxia and cellular consequences.

## 2. Physiological Characterization

**Key features of cyclic hypoxia.** The pathophysiology underlying cyclic hypoxia has been reviewed previously [4,5,6,7]. Here, we provide an historical overview of key features of cyclic hypoxia. The idea that cyclic hypoxia might exist was first described in a classic paper by Yamaura [8]. They found that tumor regrowth occurred toward the edge of window chamber tumors after irradiation. They had assumed that the tumor edge would not be hypoxic, but instead observed unstable blood flow and episodes of transient vascular stasis in the periphery. They suggested that the periphery might therefore be experiencing transient hypoxia leading to temporary radioresistance. In addition, Martin Brown demonstrated that transient radiobiologically significant hypoxia could occur, using the EMT6 tumor model [9]. This model is unique in that cells extracted from irradiated tumors are grown in vitro to generate survival curves. The terminal slope of the in vivo derived survival curve was parallel to that of in vitro hypoxia demonstrating that a proportion of cells were hypoxic in vivo. When all the hypoxic cells in tumors were killed with a hypoxic cytotoxin and tumors irradiated 24 h later, they still observed a radioresistant subfraction. This shows that tumor cells that were aerobic when drug was administered were hypoxic 24 h later when tumors were irradiated. In summary, the work of Yamaura suggested that cyclic hypoxia could occur over periods of a few minutes, whereas the work of Brown showed that this could also occur on a timescale of several hours to a day; both were likely correct.

Chaplin and Durand administered the DNA binding dye, Hoechst-33342, intravenously, to tumor-bearing mice and then isolated tumor cells and subjected them to flow cytometric analysis and assessment of clonogenic survival [10]. The more brightly Hoechst-33342 stained cells were closer to microvessels. When dye was administered intravenously during irradiation, brightly stained cells were more radiosensitive than dimly stained cells, suggesting that brightly stained cells were more aerobic. However, when dye was administered 20 min before radiation, there was no difference in radiosensitivity between dimly and brightly stained cells. The conclusion was that the cycle time for transient hypoxia was around 20 min. They theorized that temporary vascular stasis was responsible for cyclic hypoxia. However, other data does not completely support this theory, as outlined below.

**Characterization of rapid (a few cycles per hour) vs. slow cycles (cycles over hours to days).** Invasive probes or imaging methods have been used to measure parameters related to cyclic hypoxia (Table 1) [11,12,13,14,15,16,17,18,19,20]. When observation periods are between 1 and 2 h, the dominant rapid fluctuation frequencies have been 2–3 cycles per hour as assessed by Fourier transform analysis [21]. This is the same 20 min timeframe reported by Chaplin and Durand [10]. However, studies conducted in skin fold window chambers revealed that the incidence of total vascular stasis was low (<5%) [22]. Kimura et al. performed simultaneous measurements of red cell flux and perivascular oxygen concentration within individual microvessels of window chamber tumors [23]. Fluctuations in red cell flux and perivascular pO_2_ were coordinated at a frequency of 2–3 cycles per hour. A later study showed that pO_2_ several cell layers away (approaching the diffusion distance of oxygen-150 μm) from surrounding microvessels coordinated with fluctuations in red cell flux [24]. The link between variations in vascular pO_2_ and tissue pO_2_ in skin-fold window chamber tumors has also been reported using optical methods [25]. In summary, current evidence supports the theory that variations in red cell flux or hemoglobin saturation are mainly responsible for cyclic hypoxia. One cannot rule out that vascular stasis occasionally occurs, but it is not dominant.

The methods described in Table 1 that are amenable to clinical use are designated by a “*” next to the name. We have discussed the advantages and disadvantages of these methods to measure hypoxia in other reviews [26,27]. Here we focus on relative ease of use to measure cyclic hypoxia. Recessed tip polarographic microelectrodes are not commercially available. Nevertheless, studies performed with these electrodes represent the most detailed early accounting of cyclic hypoxia. The Oxylite Optical probe was used pre-clinically, including canine cancers [28,29], but its use in the clinic is challenging. Placement of this probe into human tumors would require imaging and either a physician or nurse to avoid traversing critical normal tissues and verify intra-tumoral placement [30]. These challenges, along with the limitations of requiring direct access to the tumor for placement played an important role in the cessation of the commercial Eppendorf pO_2_ electrode. Phosphorescence lifetime imaging has not been used clinically to our knowledge. Optical spectroscopy has been used in women with breast cancer [31]. This method yields real time data on two parameters related to oxygen transport—namely, total hemoglobin and hemoglobin saturation. Optical spectroscopy has not been used to measure cyclic hypoxia in human patients, but it has been used in a pre-clinical model for this purpose [32]. Optical spectroscopy is non-invasive and easy to implement. Preclinical devices are commercially available (http://www.zenalux.com/). Depth of light penetration limits utility of optical spectroscopy in human tumors. However, photoacoustic imaging enhances depth of detection by measuring emitted acoustic signals when tissue exposed to light. Photoacoustic imaging was used to monitor changes in oxygenation after radiotherapy (RT) in head and neck PDX models [33]. PET imaging of hypoxia marker drugs, such as 18-F Misonidazole, has been used in many human clinical trials to measure tumor hypoxia. One paper examined the spatial stability of PET uptake in head and neck cancers, by comparing spatial patterns over a 72 h window. A subset of patients showed instability in size or location of marker avid zones [14]. BOLD-MRI has been used extensively in pre-clinical and clinical studies, and is described in more detail below. EPRI has been used to measure cyclic hypoxia in pre-clinical models [34], but this method is not yet clinically available. However, EPRI is in early stages of commercial development for pre-clinical imaging (https://www.o2map.com/about). EPR has been used to measure kinetics of reoxygenation in pre-clinical models, but it has not been used to measure cyclic hypoxia. The technology is not widely available.

**Figure 1:**
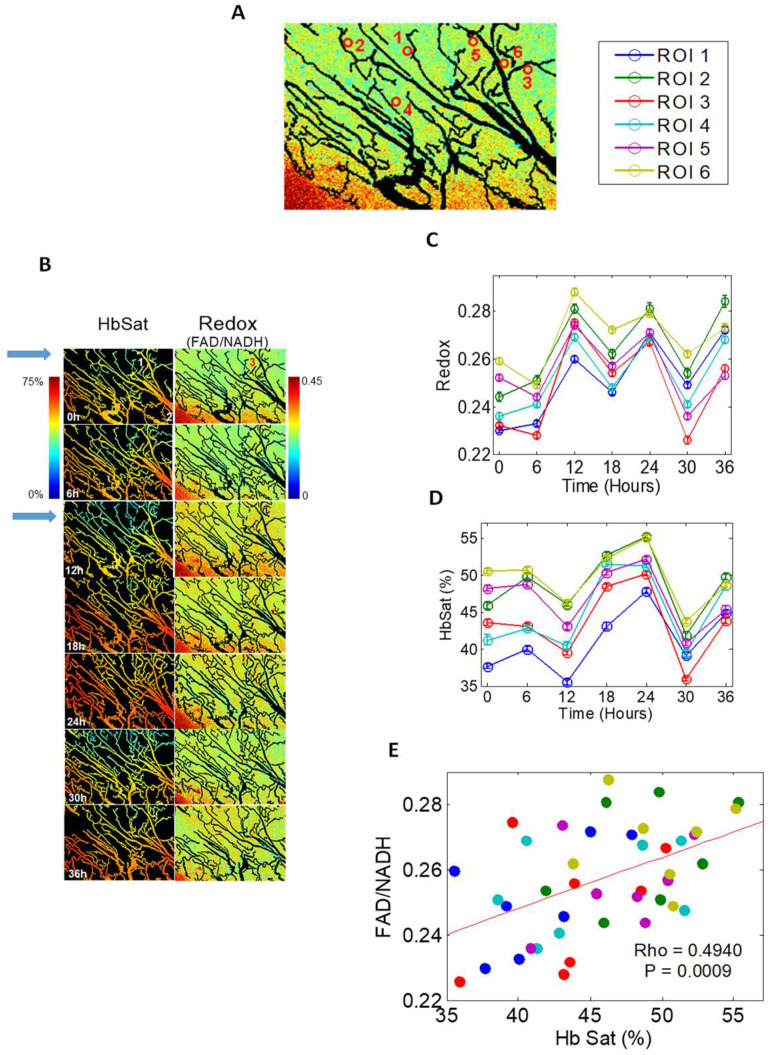
Window chamber data illustrating relatively slow fluctuations in tumor oxygenation and redox ratio. In this study, nude mice with window chambers were transplanted with FaDu head and neck cancer cells. After several days of growth, hemoglobin saturation Hb_sat_ and redox ratio (ratio of fluorescence intensities of FAD and NADH) were measured every 6 h for 36 h. (**A**) Regions of interest selected for analysis. (**B**) Paired Hb_sat_ and Redox ratios from one tumor. Note particularly the region indicated by the blue arrow at 0 and 12h. This region shows fluctuation in both parameters around a small network of microvessels. (**C**,**D**). Time course of changes in Redox ratio and Hbsat. (**E**) Over all tumors studied, there was a linear relationship between these two parameters, illustrating that there is a link between oxygen delivery and demand. In the analogy to tides and waves in Figure 2, these slow fluctuations are the tides. Panels A–D are from Skala et al. [13]. (DOI: 10.1117/1.3285584) CC-BY. Panel E is derived from the data for Panels A–D, but has not been previously published.

The skin-fold window chamber model has been used to examine slower cycle times [13]. Hemoglobin saturation and redox ratio, the ratio in inherent fluorescence intensities between flavin adenine nucleotide (FAD) and nicotinamide adenine dinucleotide (NADH) were measured every 6 h for 36 h. FAD is the main electron acceptor and NADH is the electron donor in oxidative phosphorylation—FAD/NADH correlates with oxygen demand. Coordinated fluctuations of FAD/NADH and hemoglobin saturation, were observed around networks of microvessels, suggesting that cycling was occurring in networks of blood vessels (Figure 1).

The hypoxic fraction of cervix cancer cells (SiHa) was studied as a function of time after the hypoxia marker drug, pimonidazole, was added to the drinking water of mice bearing these tumors [20]. The pimonidazole-labeled fraction, measured by flow cytometry of cells removed from tumors, increased over 96 h. They administered a second hypoxia marker drug 3 h before tumor removal. The fraction of cells labeled with the second drug was much lower than that seen for pimonidazole after 96 h exposure. These results strongly suggest that cyclic hypoxia is occurring throughout much of a tumor, but at different spatiotemporal locations.

**Figure 2:**
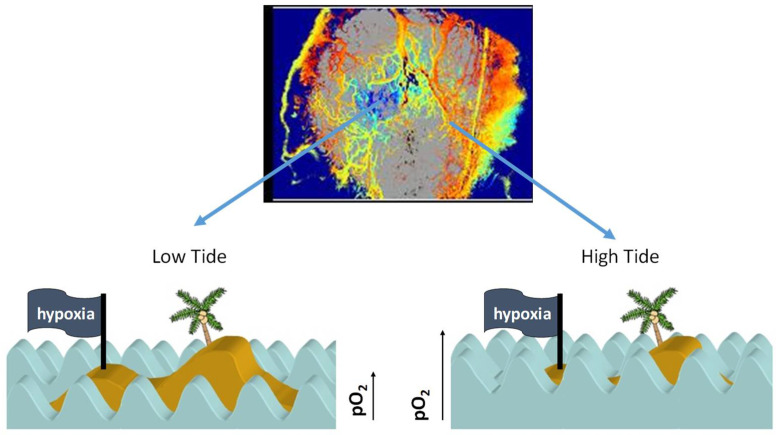
Tides and waves provide an analogy to the kinetics of cycling hypoxia. As depicted in Figure 1, the slow (quasi-steady state) fluctuations in tissue pO_2_, that occur over hours are analogous to tides. (**Upper** panel). Map of hemoglobin saturation across a tumor growing in a window chamber (blue = low saturation, orange to red = high saturation). The faster cycles, occurring 2–5 times per hour are analogous to waves. (**Lower Left**) In a region where the steady state pO_2_ (tide) is low, the higher frequency fluctuations (waves) in oxygen delivery will lead to fluctuations in the extent of tissue hypoxia, depicted by the island of hypoxia. (**Lower Right**) When the overall oxygen field is high, the waves have less of an influence on the extent of cycling hypoxia. Reproduced from Dewhirst [4], with permission from the publisher @ 2021 Radiation Research Society.

In summary, the kinetics of cyclic hypoxia are complex. Slow fluctuations occur over hours to days, and rapid fluctuations occur in the range of 2–5 cycles per hour [21,35]. We theorize that both are happening simultaneously. We previously used an analogy of tides and waves in the ocean to explain this complex kinetics [4] (Figure 2).

**Spatiotemporal distribution of cyclic hypoxia**. If cyclic hypoxia were occurring in isolation in individual microvessels, as might occur if there were temporary vascular stasis, then one would see very small regions of temporary hypoxia that are not spatially coordinated. However, this does not appear to be the case. Evidence that networks of blood vessels are coordinated in cycles of hypoxia come from two different sources of data: (1) skin fold window chambers and (2) MR/EPR imaging [18].

Spatiotemporal fluctuations of pO_2_ were studied in three different skin fold window chamber tumor models, using an oxygen sensitive Pd-porphyrin dendrimer that measures pO_2_ based on phosphorescence lifetime [12]. The dominant frequencies were between 10–40 min per cycle. All tumor types showed spatial autocorrelation of cycling, as assessed by watershed segmentation. However, regions showing autocorrelation moved over time. A similar spatiotemporal pattern of pO_2_ fluctuations in the window chamber was observed, using an oxygen sensitive nanoparticle (Supplemental Video S1) [25].

Baudelet observed fluctuations in serial MR measurements of R_2_* in mice with flank tumors [36]. R_2_* is sensitive to concentration of deoxyhemoglobin. The fluctuation patterns tended to be either random (likely machine noise) or they showed large, coordinated fluctuations amongst neighboring voxels. These results support that cyclic hypoxia occurs in networks of microvessels. Similar results were obtained by others [15,18,34].

## 3. Does Cyclic Hypoxia Occur in Canine and Human Tumors?

**Rapid cyclic hypoxia.** Fluctuation in perfusion [37] and pO_2_ have been seen in human and canine tumors [29], respectively, with cycle times similar to that seen in rodent tumors. A recent study in a limited series of patients with head and neck cancer showed fluctuations in BOLD MRI R_2_* in tumor sub-regions, particularly in involved lymph nodes [38]. The incidence ranged from 2–15% of lesion volume. Additional studies can establish the prevalence and clinical relevance in other human tumors.

**Cycles over days.** Temporal variation in intratumoral distribution of hypoxia as assessed with the 18-F labeled PET hypoxia marker drug, misonidazole, occurred over 72 h in head and neck cancer [39].

**Immunohistochemical methods.** Varying extents of mismatch between hypoxia-dependent proteins HIF-1α, CA-IX, and the well-established hypoxia marker drug, pimonidazole, have been reported in human cervical cancer xenografts and human patients with cervix cancer [40,41,42]. Ellingson et al. reported no correlation between HIF-1α and CA-IX, or uptake of pimonidazole [40]. These authors concluded that these endogenous hypoxia marker proteins would not be useful for assessment of hypoxia. In two other papers, CA-IX expression correlated significantly with pimonidazole uptake in preclinical models and a clinical series [41,42]. However, HIF-1α expression remained poorly correlated with pimonidazole uptake. One explanation for mismatch between different markers may be cyclic hypoxia. The kinetics of induction and degradation of HIF-1α are dynamic, because of the efficient post-translational degradation of the protein in the presence of oxygen [41,42]. Once HIF-1α stimulates translation of target proteins, it takes many hours to days for the proteins to reach maximal expression [41,43]. If one observes expression of a HIF-1α target, such as CA-IX, in the absence of HIF-1α itself, one conclusion is that the region must have been hypoxic sometime prior to the discernable upregulation of CA-IX, but was not hypoxic at the time of biopsy. The exception to this conclusion may reside in perinecrotic areas, where decreased HIF-1α expression is associated with nutrient depletion [41]. Cyclic hypoxia has been associated with hyper- expression of HIF-1 dependent proteins such as CA-IX [44]. This may occur because cyclic hypoxia is associated with increased oxidative stress [45]. Oxidative stress can increase HIF-1α expression, even in normoxia [46] by inhibiting degradation of HIF-1α by prolyl hydroxylases [47].

Mismatch between distribution of bioreductive hypoxia marker drugs has also been used to quantify hypoxia dynamics or cyclic hypoxia in pre-clinical model tumors [20,48]. Some of these drugs are approved for human use, thereby justifying feasibility of use in humans. One has to be cognizant of the limitations of biopsy-based methods, however. They represent very small samples of a tissue that contains distinct heterogeneity in both chronic and cyclic hypoxia. Random samples may not reflect the character of the whole tumor.

## 4. Mechanisms Underlying Cyclic Hypoxia

**Rapid cyclic hypoxia.** The fact that the dominant periodicity is around 2–5 cycles per hour, across multiple tumor types (including canine and human cancers) and methods of measurement [12,15,18,19,21,25,29,32,34,35,36,37,38,49], suggests that cyclic hypoxia results from an underlying physiologic process. Adding to this argument is the observation that normal muscle shows the same 2–3 cycle per hour of arteriolar diameter fluctuation, perfusion and pO_2_ [21,22]. The question that arises then, is what physiologic process can affect perfusion and pO_2_ in normal tissue with cycle times of 2–5 cycles per hour? Both heart and respiratory rates occur at much higher frequencies than oscillations seen with cyclic hypoxia [50]. Arteriolar vasomotion in the range of 30–60 cpm occurs in microcirculatory networks in vivo [51,52]. This frequency is much too fast to be consistent with cyclic hypoxia. It has been speculated that sleep apnea could contribute to cyclic hypoxia, but this does not occur in awake subjects or in dogs who have been intubated for measurements [6]. Yet, cyclic hypoxia occurs in tumors of both species.

We theorize that thermoregulation may be the physiologic factor that drives cyclic hypoxia. The body is equipped with sophisticated means to transport heat, via circulation from body core to periphery, where active heat exchange occurs across the skin surface [53]. Transient receptor potential (TRP) receptors signal the brain to control vascular response to changes in temperature [54,55,56]. TRP receptors are located throughout the body [54,57,58]. Receptors associated with sensing temperature and thermal regulation from elevated temperature include TRPV1, TRPV2, TRPV3, and TRPV4 [59]. Knockout of TRPV1 in mice compromises their ability to thermoregulate [54]. The brain responds to changes in systemic temperature and TRPV signaling by increasing neuronal activation in the preoptic region of the hypothalamus [56]. The time course for these responses is in the range of 20 min. However, these responses can be seen with local change in temperature as well. The kinetics of tissue response to local thermal stress were previously examined in canine muscle [60]. To elicit a thermoregulatory response, a microwave applicator was used to apply power to muscle. They observed oscillations in muscle temperature, with a frequency in the range of 2–3 cycles per hour. Goncalves et al., performed serial R_2_* measurements in tumors of mice, while simultaneously measuring muscle arteriolar pO_2_, using a pulse oximeter [15]. The kinetics of muscle pO_2_ fluctuations (2–3 cycles per hour) mirrored that of the R_2_* oscillations in tumors in the same animals. They presumed that pulse oximeter measurements were reflective of oscillations in systemic pO_2_. However, arteriolar pO_2_ of muscle is much lower than the blood gas pO_2_, the result of longitudinal gradients [61]. It is more likely that fluctuations in muscle pO_2_ were due to perfusion cycles caused by thermoregulation. Consistent with thermoregulation theory, we have observed a slower arteriolar vasomotion kinetic, consistent with a cycle time of 20–30 min in window chambers [22]. However, additional studies are required to prove whether thermoregulation is an underlying cause for cyclic hypoxia. Although TRP receptor expression is ubiquitous throughout the body, we do not know whether these receptors are involved in thermoregulation in deep-seated tissues. There is no question, however, that TRP receptors are very important for thermoregulation in skin and muscle. Since the vast majority of preclinical cyclic hypoxia studies involved tumors growing in peripheral tissues, existing data may overestimate the importance of this phenomenon in deep-seated tumors. New studies of deep-seated tumors are essential for understanding this important question.

**Slow cycles occurring over hours to days.** The mechanism underlying slower kinetics of cyclic hypoxia remain undefined. It is speculated that this may be the result of vascular remodeling within the tumor [5]. To fully understand mechanisms for this slower kinetic behavior, further research is required.

**Differences in fluctuation magnitude.** There are large differences in fluctuation magnitude between different tumor types and sites of tumor growth [35,62]. Others have observed that the fluctuation magnitudes are associated with the presence of vascular smooth muscle in tumors, which presumably would be more vasoactive [63]. More work is needed to uncover mechanisms associated with such variations.

## 5. Cellular Consequences

As previously discussed, there are significant challenges in accurately measuring the parameters associated with cyclic hypoxia. As a consequence, although it is clear that the biological response to cyclic conditions differs to chronic hypoxia, the breadth of conditions and models used makes comparing studies difficult. In particular, few studies use cycling conditions between physiologically relevant levels of oxygen while most incorporate periods of reoxygenation to 21% oxygen (air) (Table 2). However, despite this, many common findings have emerged and will be discussed.

**The contribution of reactive oxygen species (ROS).** ROS is a broad term used to describe reactive chemical species containing oxygen, typically generated during cellular metabolism. Two key mechanisms for ROS generation are enzymes that produce ROS as a by-product, and electrons that “leak” from the electron transport chain (ETC) and subsequently interact with oxygen to form ROS [110,111]. As reactive molecules can perturb normal cellular function, it is imperative that mechanisms exist to tightly regulate ROS levels. These mechanisms include the ability to quench reactivity through antioxidants such as glutathione and enzymes including superoxide dismutase (SOD). Failure to restrict ROS levels causes oxidative stress. Oxidative stress is defined as conditions where potentially lethal damage to proteins, lipids, and DNA occurs in a ROS-dependent manner [112,113]. However, ROS also play essential roles and have been implicated in cellular signaling pathways critical to proliferation, host cell defense, autophagy, and stem cell differentiation [45,112,114,115,116,117]. Somewhat paradoxically, despite an insufficient supply of oxygen, ROS have been shown to increase in chronic hypoxia. Specifically, hypoxia-induced ROS have been attributed to mitochondrial disfunction, resulting in more electrons being released from the ETC [118,119,120]. In addition, the expression of enzymes including NADPH oxidase 4 (NOX4), which produce ROS as a by-product of their activity, are induced in hypoxic conditions and contribute to ROS levels [111,121]. Less surprisingly, single reoxygenation events (usually to 21% O_2_) have been well-documented to induce ROS through NOX, nitric oxide synthase (NOS), and xanthine oxidase (XO) signaling, in addition to further damage to the ETC [111,122]. Importantly, cyclic hypoxia both in vitro and in vivo has been shown to increase ROS levels [65,66,70,72,73,74,75,76,99]. An increase in the mRNA and protein expression of NOX and NOS has been demonstrated in conditions of cyclic hypoxia [69,70,73,74,75]. Most importantly, when compared directly, ROS have been shown to increase in cyclic hypoxia to a greater extent than in chronic hypoxia [72,73,74,76]. To support this observation, levels of NOX4 mRNA and protein were measured in cyclic and chronic hypoxia in glioblastoma cell lines in vitro and found to increase to a greater extent in cyclic hypoxia [73,74]. Furthermore, knockdown of specific NOX proteins both in vitro and in vivo reduced ROS levels in cyclic hypoxia [70,73,74].

As mentioned previously, oxidative stress results from elevated cellular ROS levels and can lead to a loss of viability [112,113]. In vitro and in vivo models of cyclic hypoxia have shown increases in a variety of oxidative stress markers, including increased expression of p22phox and NRF2 [99,100], and the presence of DNA damage [64,67,100,101]. DNA damage induced as a result of oxidative stress includes the accumulation of aberrant DNA bases such as 8-oxoguanine. An increase in 8-oxoguanine in cyclic hypoxia has been observed in vivo, although this appears to be dependent on the mouse model used [100,101,102]. Furthermore, in vitro models of cyclic hypoxia have detected a significant increase in DNA damage by comet assay compared to normoxia [64,67]. The transcription factor, NFκB, plays a key pro-survival role in response to oxidative stress [112,123]. Multiple studies have observed increased activation of NFκB in cyclic hypoxia determined by phosphorylation status and increased expression of NFκB-target genes [90,95,99]. However, despite the activity of NFκB, colony survival assays conducted in cyclic hypoxia in colorectal, breast, and glioblastoma cell lines demonstrated a loss in viability that is ROS-dependent [65,66]. The mechanism of cell death in cyclic hypoxia is unclear and likely multi-factorial. Interestingly, it has been shown that the decrease in cell viability in cyclic hypoxia can be exacerbated through treatment with chloroquine, an inhibitor of autophagy. Autophagy is thought to protect cells in cyclic hypoxia through the promotion of mitophagy which would decrease ROS produced through the ETC [66].

**The HIF-1 response to cyclic hypoxia.** The hypoxia inducible factor 1 (HIF-1) transcription factor is formed from the binding of a HIF-1α and HIF-1β subunit. In normoxic conditions the HIF-1α subunit is hydroxylated by members of the prolyl hydroxylase domain (PHD) family. Hydroxylation targets HIF-1α for proteasomal degradation mediated by the E3 ligase, von Hippel Lindau (VHL). In the absence of VHL-mediated degradation, HIF-1α binds to HIF-1β and regulates the transcription of a variety of effector genes with diverse functions including metabolism, angiogenesis, growth, and apoptosis [124,125]. Importantly, HIF-1α accumulates in a wide variety of oxygen concentrations ranging from <0.1 to 3% O_2_ [126,127,128]. More recently, the alternative α subunits HIF-2α and HIF-3α have been identified and shown to bind HIF-1β [129,130]. A number of in vitro studies have demonstrated that HIF-1α levels are higher in cyclic hypoxia compared to chronic exposure and that the increased levels of HIF-1α correlate with transcriptional activity and expression of downstream HIF-1 target genes [69,70,72,76,79,87,89,97]. An in vivo study also demonstrated that cyclic hypoxia induces the expression of a variety of HIF-1 target genes in orthotopically grown cervical tumors in mice [44]. Interestingly, though the stabilization of HIF-2α increases in chronic hypoxia, stabilization in response to cyclic hypoxia was shown to decrease in vitro and in vivo [97,107,108,127].

The role of ROS in mediating HIF-1α stability through inhibition of the PHDs has been well-described (reviewed in [131,132,133]). These data support the hypothesis that increased ROS levels in cyclic hypoxia contribute to HIF-1α stabilization [118,134,135]. However, it should be noted that these studies all used cyclic conditions which included periods of 21% O_2_, which are non-physiological, and also likely to induce significant ROS (Table 2). In contrast, a single study has investigated HIF-1α stabilization in cycling conditions which featured physiologically relevant levels of oxygen. In this case, oxygen tensions were rapidly cycled every 5 min between <0.1–7.76% O_2_ and found that while HIF-1α expression increased in cyclic hypoxia, it accumulated to a lesser extent in cyclic compared to chronic hypoxia [87]. It is clear that in the studies demonstrating increased HIF-1α in cyclic hypoxia this was ROS-dependent as the use of scavengers reduced HIF-1α accumulation [72,73,75,99]. However, it remains unclear whether cycling between physiologically relevant levels of hypoxia leads to an increase in ROS and whether this, in turn, contributes to increased HIF-1α stabilization. Another, non-mutually exclusive explanation for the increased HIF-1 mediated activity observed in cyclic hypoxia, is the contribution of a non-nuclear HIF-1 response. In response to cellular stress, including hypoxia, the cell packages key mRNA transcripts associated with the stress response into cytoplasmic compartments known as stress granules [136]. It has been shown that, upon reoxygenation (21% O_2_), these stress granules disaggregate and their contents are released to amplify HIF-1 downstream signaling [46].

Due to the biological complexity of HIF stabilization and signaling, mathematical modeling has emerged as a useful way to increase our understanding of HIF biology. Though HIF dynamics in response to cyclic hypoxia have not been explicitly modeled, this strategy represents a promising way to decipher biological responses to complex variables such as oxygen concentration and cycling frequency without the need to test every condition experimentally. By reducing the complex system that controls HIF-1 regulation to a simpler “core” system, existing biological knowledge of the interplay of the components within the core system can be used to inform the model to make predictions on situations that have not been experimentally tested [137]. For example, oxygen tensions in different regions of a spheroid were measured with intracellular probes that were subsequently used to inform the parameters of a mathematical model. A spheroid was then subjected to an in silico decrease in oxygen concentration from both 8 to 3% O_2_ and from 21 to 1% O_2_ and HIF-1 activity was modeled over time. The model predicted that cells on the periphery of the spheroid would have the highest level of HIF-1 signaling once exposed to hypoxia even though the core of the spheroid was initially the most hypoxic region. This somewhat counterintuitive finding was hypothesized to occur due to the relative speed of the change in oxygen concentration at the periphery of the spheroid relative to the hypoxic core, where the faster change results in a larger initial accumulation of HIF-1 [138].

In addition to investigating the HIF response, mathematical models have been used to predict cellular characteristics in the face of a dynamic oxygen environment. For example, a mathematical model was created that included a population of cells with a continuous gradient of metabolic potentials ranging from fully oxidative to fully glycolytic to mimic the metabolic variation present in tumors. The values of these variables were used to inform the model as to the distribution of oxygen across the tumor and where regions of cyclic hypoxia would arise. Analysis was then carried out to determine how the subpopulations interacted with one another under the given oxygen conditions. One of the key findings from this approach was that, in conditions where the oxygen level tended to fluctuate, a higher phenotypic variation became a competitive advantage in terms of managing oxygen consumption in a changing environment [139]. These studies all further the notion that distinct biological signaling occurs depending on the oxygen dynamics present in the tumor and that mathematical modeling is a useful tool to dissect the complex contribution of the dynamic oxygen environment.

**Radiotherapy.** The increased radioresistance of hypoxic tumors/cells has been well-characterized and is known to contribute to patient prognosis. The oxygen enhancement ratio (OER) describes the relative radiosensitivity of cells in hypoxic versus normoxic conditions and demonstrates that hypoxic cells can be 2.5–3 times more radioresistant than their oxygenated neighbors [140,141,142]. The requirement for oxygen at the time of irradiation in order to induce maximum levels of DNA damage has been reviewed recently [143]. Particularly relevant here are the multiple studies that demonstrate that exposure to different chronic and cyclic hypoxic treatment schedules prior to irradiation impact radiosensitivity. Clonogenic assays show that cells pre-treated with cyclic hypoxia are more radioresistant when compared to cells pre-treated with chronic hypoxic [71,72,74,77,91,92,93]. This has also been confirmed in vivo where glioblastoma tumors irradiated in mice exposed to cyclic hypoxia were more radioresistant than the chronic hypoxia controls [72]. These data demonstrate that the OER can be affected by a biological component, distinct from the oxygen concentration at the moment of irradiation, and suggest that this biological component could be influenced by the nature of prior hypoxia exposure. Notably again, the majority of these studies were conducted in cycling conditions that included periods of reoxygenation to 21% O_2_ and, therefore, oxidative stress-induced DNA damage. It is, therefore, surprising that the pretreatment with these cyclic conditions did not increase radiosensitivity, suggesting a selection pressure to increase cell viability.

The contribution of HIF-1 to the radiosensitivity of cells in hypoxia has been investigated and broadly demonstrates that inhibition or loss of HIF-1 increases radiosensitivity and is likely attributable to the impact on tumor vasculature, metabolism and cell cycle control [46,72,144,145,146,147]. However, it is not surprising, given the diverse and numerous targets of HIF-1, that a context dependent balance between HIF-1-mediated radiosensitivity and radioresistance is likely [148,149]. As previously mentioned, some studies have suggested that HIF-1 is more active in cyclic conditions compared to chronic hypoxia and, therefore, the impact on radiosensitivity could also be more significant. In support of this hypothesis, in vitro and in vivo experiments determined that radioresistance was increased in cyclic hypoxia and that this was dependent on HIF-1 signaling [71,72,77].

The eukaryotic translation initiation factor 2α (eIF2α) has been identified as a factor that influences the OER in hypoxia including cyclic conditions [65]. eIF2α is typically associated with the unfolded protein response (UPR) though it also plays a role in regulating the expression of enzymes involved in the synthesis of key antioxidants such as glutathione and cysteine [150]. Inhibition of eIF2α was shown to increase radiosensitivity in vivo and, although not directly measured or induced, the presence of cyclic hypoxia was inferred from analysis of pimonidazole positive and negative regions [65]. Interestingly, radiosensitization only occurred when eIF2α was inhibited prior to radiation and was attributed to an eIF2α-dependent role in mediating the cellular response to ROS. Inhibition of eIF2α prior to irradiation renders the cell incapable of managing the increased ROS generated by irradiation-induced reoxygenation. Supportive in vitro experiments demonstrated that cell death caused by cyclic hypoxia increased further when eIF2α was inhibited, and that this was ROS-dependent. Furthermore, enzymes involved in the creation of glutathione and cysteine were shown to increase in cyclic hypoxia in an eIF2α-dependent manner [65]. Similarly, SLC25A10, SLC25A1, and GOT1 which have roles in mitigating oxidative stress, are induced in response to cyclic hypoxia and contribute to radioresistance [91,92,94]. Interestingly, a recent report describes the use of Angiotensin II type 1 receptor blockers as a means of improving radiation response through the inhibition of transient hypoxia [151].

**Chemotherapy.** Hypoxic tumors demonstrate increased chemoresistance due to a variety of factors including: large diffusion distances from blood vessels, decreased cellular drug uptake, decreased apoptosis, altered pH, and decreased proliferation [1,152]. Many of these chemoresistant features are mediated by HIF-1 signaling [152,153]. Recently, increased drug efflux and apoptotic resistance have been studied in the context of cyclic hypoxia and found that chemoresistance exceeded that of cells treated in chronic hypoxia [76,83].

In cyclic hypoxia, a HIF-1 dependent mechanism results in the upregulation of the cell membrane efflux pump, ATP Binding Cassette Subfamily B Member 1 (ABCB1). Cells in cyclic hypoxia demonstrated an increased capacity to efflux the chemotherapeutic drug Rh123 compared to cells in chronic hypoxia in an ABCB1-dependent manner. This ability to efflux chemotherapy drugs in cyclic hypoxia translated into increased resistance of glioblastoma cells to doxorubicin and carmustine (BCNU) in vitro [83]. Cells in cyclic hypoxia also demonstrate an increased ability to resist apoptosis in response to chemotherapeutics. The anti-apoptotic gene, B-cell lymphoma extra-large (Bcl-xL), was shown to be upregulated to a greater extent in cyclic hypoxia compared to chronic hypoxia in a HIF-1 dependent manner in glioblastoma and medulloblastoma cell lines [76,85]. Treating cells in cyclic hypoxia with temozolomide (TMZ) combined with Bcl-xL inhibition resulted in increased cell kill compared to TMZ alone. Interestingly, Bcl-xL upregulation was also found to occur in a ROS-dependent manner in cyclic hypoxia. Consequently, in vivo ROS scavenging with Tempol increased the effectiveness of TMZ treatment [76]. Furthermore, exposure to a course of cyclic hypoxia spanning 50 days led to selection for loss of the p53 tumor suppressor gene. Loss of p53 translated into increased survival of sub clones from the cells treated in cyclic hypoxia compared to normoxia when treated with the chemotherapeutic etoposide [96].

**Figure 3:**
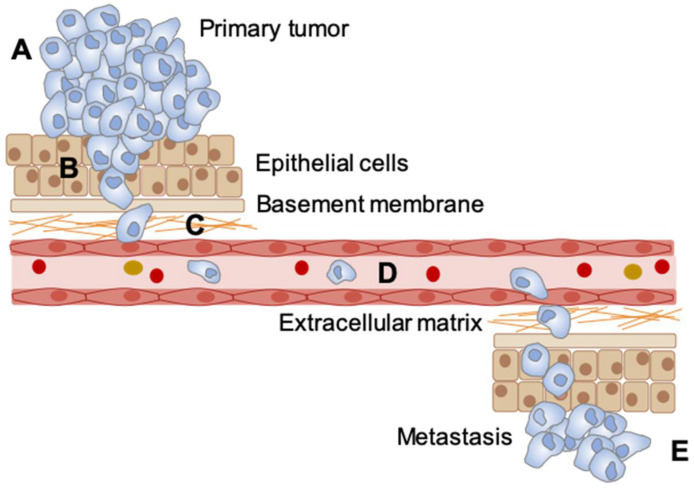
The effect of cyclic hypoxia on different stages in metastasis. (**A**) number of studies have demonstrated that exposure to cyclic hypoxia drives metastasis and importantly, that this occurs at all the key stages of the process. A. EMT/stemness [79,80,81,85,86,88]. (**B**) Invasion/migration [78,80,81,82,84]. (**C**) Modulation of the extracellular matrix (ECM) [160]. (**D**) Survival in blood circulation [89]. (**E**) Proliferation in target organ [86,89,99,103,104,105,106,159].

**Metastasis.** Understanding the processes of metastasis is essential to improving cancer therapy, as the vast majority of cancer-related deaths occur as a result of tumor spread/metastasis [154]. In order to metastasize, cancer cells undergo a series of rate-limiting steps including: the epithelial to mesenchymal transition (EMT), invasion and migration through the basement layer, survival in blood circulation, and proliferation at the second site [155,156,157]. The tumor microenvironment has been shown to contribute to multiple steps in the progression of metastasis [158]. Hypoxia in particular aids cancer cells in completing these steps primarily through HIF-1 signaling [156,157]. Utilizing regulated gas supplies to modulate the oxygen level in the environment of mice with xenograft tumors, it was demonstrated that mice treated with cyclic hypoxia develop more metastasis than mice exposed to normal oxygen conditions [86,89,99,103,105,106,159]. In addition, studies that have compared the ability of tumors to form metastasis in xenograft mice treated with cyclic and chronic hypoxia show that tumors from mice treated with cyclic hypoxia have more metastasis [86,89,103,159]. The effect of cyclic hypoxia on the cellular abilities required to successfully metastasize are summarized in Figure 3.

**Metabolism.** Cellular metabolism is known to be altered in cancers and is a hallmark of cancer. A major factor that influences the metabolic shift in tumors is the stabilization of HIF-1α in response to hypoxia. HIF-1 targets include genes such as GLUT1 and PDK1, which can increase glycolysis and downregulate oxidative phosphorylation, respectively. These metabolic changes allow cells to generate sufficient energy in environments devoid of oxygen [161,162]. Currently, little is known about cellular metabolism in response to cyclic hypoxia in cancer, although a wealth of literature studying cyclic hypoxia associated with obstructive sleep apnea has identified a variety of metabolic effects, including alternations in lipid and glucose metabolism (reviewed in [163,164,165]). Interestingly, one study showed that increased levels of serum triglycerides induced by cyclic hypoxia occurred in a HIF-1 dependent manner in a heterozygous HIF-1 knockout mouse model compared to the wild-type [109]. Another study measuring the expression of a panel of 30 genes involved in glucose metabolism in vitro, observed a significant increase in the expression of 15 genes contained within the panel in cyclic hypoxia [98]. Furthermore, culturing of cells in cyclic hypoxia over long time periods caused a metabolic shift that persisted in normoxia. Increases in HIF-1α and GLUT1 expression were seen in normoxia along with a significant increase in oxygen consumption [96]. Interestingly, an emerging clinical strategy to sensitize tumors to radiotherapy is through the modulation of the oxygen consumption rate of tumors prior to irradiation [166,167,168]. It is possible that these agents may lead to conditions similar to cyclic hypoxia. Further study is needed to assess the effects of cyclic hypoxia on metabolism as well as how these effects compare to those seen in chronic hypoxia.

## 6. Future Directions

It is clear, and has been for some time, that regions of hypoxia in tumors are not static; instead, oxygen levels fluctuate and cycle. Research into this phenomenon and delineating impact on tumor progression and therapy response has been limited by our ability to measure physiological meaningful parameters and then model these in vitro. Consequently, physiologically relevant signaling caused by cyclic hypoxia that could potentially inform clinical practice is poorly understood. More physiologically relevant in vitro models of cyclic hypoxia, utilizing experimental systems more capable of mimicking the complex oxygen environments found in tumors, will be instrumental in understanding the clinically relevant consequences of cyclic hypoxia. Advances in imaging and mathematical modeling present exciting methods to inform physiologically relevant oxygen conditions. Mathematical modeling in particular presents an opportunity to lessen dependence on individual in vitro models that generally lack comparability between labs, and study complex oxygen dynamics that cannot be experimentally tested. Notably, these developments are likely to significantly impact our understanding of developing therapeutic strategies, which aim to manipulate oxygen consumption within tumors as a means of reducing hypoxia and increasing radiosensitivity [166,167,168]. It is plausible that agents which inhibit oxygen consumption/mitochondrial metabolism may lead to biological consequences similar to those described in the response to cyclic hypoxia.

## Figures and Tables

**Table 1:** Summary of the cyclic hypoxia measurement techniques. PET = positron emission tomography; SPECT = single-photon emission computed tomography; BOLD MRI = blood oxygen level dependent MRI, measured by R_2_ *, OE-MRI measures R1, which is sensitive to [O_2_] in tissue; EPR = electron paramagnetic resonance; EPRI = electron paramagnetic resonance imaging; * = methods that have been used to measure hypoxia in human tumors and are amenable to measuring cyclic hypoxia. Rapid cycles = cycles occurring over 1–2 h observation. Slow cycles = cycles occurring over many hours to days.

Measurement Techniques	Requires Chemical Probe	Direct pO_2_ Measurement	Frequency of Detection	Spatial Resolution	Ref.
**Polographic O₂ microelectrodes**	No	Yes	<<1 s Suitable for rapid cycles	20–30 μm	[11]
**Oxylite optical probe**	No	Yes	<<1 s Suitable for rapid cycles	250 μm	[11]
**Phosphorescence lifetime imaging**	Pd-porphyrin dendrimer, IV	Yes	2–2.5 min Suitable for rapid cycles	<mm-cm	[12]
Optical Spectroscopy *	No	No	30 s Suitable for rapid and slow cycles	mm-cm	[13]
PET/SPECT *	Yes	No	>24 h Suitable for slow cycles	Several mm	[14]
BOLD MRI *	No	No	4 s Suitable for rapid cycles	Sub mm-mm	[15]
OE-MRI *	No	No	2.5 min Suitable for rapid cycles	0.24 mm	[16]
**EPR**	Implantable O_2_ sensitive spin probe with resonator	Yes	10–60 s Suitable for rapid and slow cycles	mm-> cm	[17]
**EPRI**	O_2_ sensitive spin probe, IV	Yes	2–3 min Suitable for rapid and slow cycles	1.6–1.8 mm	[18]
**F-19 MRI**	Intratumoral injection of hexafluorobenzene	Yes	1.5 min Suitable for rapid and slow cycles	2 mm	[19]
Dual hypoxia marker drugs *	2 hypoxia marker drugs given at various intervals from each other	No	These probes average extent of hypoxia over 60–90 min	μm	[20]

**Table 2:** Summary of published studies describing cyclic hypoxia. Studies highlight in **grey** are in vitro studies which include reoxygenation to 21% O_2_. Studies in **blue** are those that include reoxygenation to 21% O_2_ but do not include multiple cycles i.e., a single reoxygenation event. Those in **green** are in vitro cyclic hypoxia studies that include cycling between two physiologically relevant oxygen concentrations. Finally, those in **orange** are in vivo studies and indicate the concentration of oxygen in the air the mice were given to breathe and for how long.

Cycling Conditions (% O_2_ and Length of Exposure)	Number of Cycles	Ref.
1% O_2_ (6.67 min)—21% O_2_ (6.67 min)	72	[64]
<0.02% O_2_ (1 h)—21% O_2_ (1 h)	2–5	[65]
<0.02% O_2_ (1 h)—21% O_2_ (1 h)	2–5	[66]
<0.02% O_2_ (16 h)—21% O_2_ (1 h)	1	[67]
1% O_2_ (1 h)—21% O_2_ (30 min)	4	[68]
<1% O_2_ (1 h)—21% O_2_ (30 min)	3	[69]
1% O_2_ (2 h)—21% O_2_ (2 h)	1–3	[70]
0.5–1% O_2_ (1 h)—21% O_2_ (30 min)	3	[71]
0.5–1% O_2_ (1 h)—21% O_2_ (30 min)	3	[72]
0.5–1% O_2_ (10 min)—21% O_2_ (10 min)	12	[73]
0.5–1% O_2_ (10 min)—21% O_2_ (10 min)	12	[74]
1.5% O_2_ (30 s)—21% O_2_ (5 min)	60	[75]
0.5–1% O_2_ (1 h)—21% O_2_ (30 min)	3	[76]
0.1% O_2_ (24 h)—21% O_2_ (72 h)	20	[77]
1% O_2_ (48 h)—21% O_2_ (24, 48, 72 h)	1	[78]
1% O_2_ (24 h)—21% O_2_ (24 h)	1, 5, 10	[79]
1% O_2_ (12 h)—21% O_2_ (12 h)	7	[80]
1% O_2_ (12 h)—21% O_2_ (12 h)	5	[81]
1% O_2_ (12 h)—21% O_2_ (12 h)	5, 10, 15, 20	[82]
0.5–1% O_2_ (1 h)—21% O_2_ (30 min)	3	[83]
<1% O_2_ (‘shots’ for 8, 72 h)—21% O_2_ (n/a)	1,3/week	[84]
1% O_2_ (48 h)—21% O_2_ (48 h)	18–20	[85]
1% O_2_ (24 h)—21% O_2_ (24 h)	5	[86]
<0.1% O_2_ (5 min)—7.76% O_2_ (5 min)	36 or 108	[87]
1% O_2_ + nutrient deprivation (7 days)—21% O_2_ (1–3 weeks)	1–3	[88]
0.5% O_2_ (1 h)—21% O_2_ (30 min)	3	[89]
1% O_2_ (1 h)—21% O_2_ (30 min)	4	[90]
<1% O_2_ (48 h)—21% O_2_ (120 h)	16 or 25	[91]
<1% O_2_ (48 h)—21% O_2_ (120 h)	16 or 25	[92]
<0.1% O_2_ (30 min or 3 h)—7% O_2_ (30 min)	21 or 72	[93]
<0.1% O_2_ (48 h)—21% O_2_ (120 h)	16 or 25	[94]
1% O_2_ (1 h)—21% O_2_ (30 min)	4	[95]
0.2 or 1% O_2_ (8, 16, 24 h)—21% O_2_ (8, 16, 24 h)	3, 6, 50	[96]
1.5% O_2_ (30 s)—21% O_2_ (5 min)	15, 30, 60	[97]
<1% O_2_ (8, 72 h)—21% O_2_ (undefined)	30, 60	[98]
7% O_2_ (1 h)—21% O_2_ (30 min)	3	[72]
5% O_2_ (30 s)—21% O_2_ (1 min)	180/day (21 days)	[99]
7% O_2_ (10 min)—21% O_2_ (10 min)	12	[73]
10% O_2_ (~3 min)—21% O_2_ (~3 min)	80/day (7 days)	[100]
7% O_2_ (10 min)—21% O_2_ (10 min)	12/day (5/week for 6 weeks)	[101]
7% O_2_ (10 min)—21% O_2_ (10 min)	6 or 12/day (5/week for 6 weeks)	[102]
7% O_2_ (1 h)—21% O_2_ (30 min)	3	[71]
5–7% O_2_ (10 min)—21% O_2_ (10 min)	12/day	[103]
7% O_2_ (10 min)—21% O_2_ (10 min)	12/day (21 days)	[104]
7% O_2_ (10 min)—21% O_2_ (10 min)	12	[76]
8% O_2_ (10 min)—21% O_2_ (10 min)	12/day (35 days)	[105]
8% O_2_ (10 min)—21% O_2_ (10 min)	12/day	[106]
7% O_2_ (1 h)—21% O_2_ (30 min)	3/day (1 day)	[95]
7% O_2_ (10 min)—21% O_2_ (10 min)	12/day (21 days)	[44]
5% O_2_ (15 s)—21% O_2_ (5 min)	9/day (10 days)	[97]
5% O_2_ (15 s)—21% O_2_ (5 min)	9/day (10 days)	[107]
5% O_2_ (15 s)—21% O_2_ (5 min)	9/day (10 days)	[108]
5% O_2_ (30 s)—21% O_2_ (30 s)	5 days	[109]

## Supplementary Materials

The following are available online at https://www.mdpi.com/2072-6694/13/1/23/s1, Video S1: Dynamics of cycling hypoxia in a window chamber containing a 4T1 tumor. An oxygen sensitive nanoparticle was used to directly measure pO_2_ by measuring phosphorescence lifetime. Hemoglobin saturation was measured optically. Measurements were taken every 7.5 min for 60 min. The X-Y plane represents the surface of the tumor; the Z-plane depicts pO_2_. Microvessel hemoglobin saturation is superimposed on the pO_2_ images. The spatiotemporal dynamics clearly illustrate that the changes are occurring simultaneously in many vessels. But one can see increases in some regions with decreases in other areas. This illustrates the complex hemodynamics that can occur, especially in tumor vascular networks. Data are from Palmer et al. [25].
